# Impact of KMT2A Rearrangement and CSPG4 Expression in Pediatric Acute Myeloid Leukemia

**DOI:** 10.3390/cancers13194817

**Published:** 2021-09-26

**Authors:** Lina Marie Hoffmeister, Eser Orhan, Christiane Walter, Naghmeh Niktoreh, Helmut Hanenberg, Nils von Neuhoff, Dirk Reinhardt, Markus Schneider

**Affiliations:** 1 Department of Pediatric Hematology and Oncology, University Children’s Hospital Essen, University of Duisburg-Essen, 45122 Essen, Germany; Christiane.Walter2@uk-essen.de (C.W.); Naghmeh.Niktoreh@uk-essen.de (N.N.); Helmut.Hanenberg@uk-essen.de (H.H.); Nils.vonNeuhoff@uk-essen.de (N.v.N.); dirk.reinhardt@uk-essen.de (D.R.); 2 Centre for Research Acceleration in Pediatrics GmbH, 30175 Hannover, Germany; orhan.eser@aml-bfm.de; 3 Department of Otorhinolaryngology and Head/Neck Surgery, Heinrich Heine University, 40225 Düsseldorf, Germany

**Keywords:** acute myeloid leukemia, childhood acute myeloid leukemia, pediatric, *KMT2A*, *MLL*, CSPG4, NG2, AML-BFM

## Abstract

**Simple Summary:**

In order to determine the impact of *KMT2A* rearrangements (*KMT2A*-r) on the clinical characteristics and treatment outcome of pediatric acute myeloid leukemia (AML) patients, we analyzed a German population-based AML cohort of 967 patients, diagnosed between 2004 and 2019, from which 241 harbored *KMT2A*-r. *KMT2A*-r is associated with a higher disease burden and younger age at diagnosis, as well as morphologic subtype of AML M5. The 5-year overall survival rate of patients with *KMT2A*-r was comparable to those of patients without *KMT2A*-r. When analyzing AML blasts with *KMT2A*-r for the presence of additional genetic aberrations using different methods, e.g., classical cytogenetics, next-generation sequencing and multiplex PCR, we found the frequency of *KRAS* mutations increased, whereas *FLT3*-ITDs decreased compared to patients without *KMT2A*-r. Finally, we demonstrated that a correlation between CSPG4 expression and *KMT2A*-r exists in pediatric AML blasts; however, CSPG4 expression was not specific for blasts with *KMT2A*-r.

**Abstract:**

*KMT2A* rearrangements (*KMT2A*-r) are among the most common structural aberrations in pediatric acute myeloid leukemia (AML) and are very important for the risk group stratification of patients. Here, we report the outcome of 967 pediatric AML patients with a known *KMT2A*-r status. The large cohort was characterized by morphology, multicolor flow cytometry, classical cytogenetics and mutation analysis via panel sequencing. In total, the blasts of 241 patients (24.9%) showed *KMT2A*-r. *KMT2A*-r is associated with FAB M5, a high white blood cell count and younger age at diagnosis. When subgroups were combined, *KMT2A*-r had no impact on event-free survival (EFS) and overall survival (OS); however, various subgroups showed a different prognosis, ranging from a <50% OS for *KMT2A/AFDN* (*n* = 11) to a 100% chance of survival for patients harboring the rare translocation *KMT2A/SEPTIN9* (*n* = 3, follow up of 3.7 to 9.6 years). A positive correlation of *KMT2A*-r with *KRAS* mutations (*p* < 0.001) existed, albeit without any prognostic impact. In addition, *FLT3*-ITDs were detected less frequently in AML with *KMT2A*-r (*p* < 0.001). Furthermore, *KMT2A*-r were mutually exclusive, with mutations in *NPM1* (*p* = 0.002), *KIT* (*p* = 0.036), *WT1* (*p* < 0.001) and *CEBPA* (*p* = 0.006), and translocations *NUP98/NSD1* (*p* = 0.009), *RUNX1/RUNX1T1* (*p* = 0.003) and *CBFB/MYH11* (*p* = 0.006). In the 346 patients tested for CSPG4 expression, a correlation between CSPG4 expression and *KMT2A*-r was confirmed. However, CSPG4 expression also occurred in patients without *KMT2A*-r and had no significant prognostic impact on EFS and OS.

## 1. Introduction

Pediatric acute myeloid leukemia (AML) is a heterogeneous malignancy of the hematopoietic system caused by a variety of different recurrent genetic aberrations that determine the risk group stratification and treatment of the AML patients [1,2]. The overall survival rate for pediatric AML has increased tremendously over the past few decades [3]. Currently, pediatric patients diagnosed with AML have a 5-year overall survival (OS) of 76 ± 4%, and a 5-year event-free survival (EFS) is observed in 50 ± 2% of the patients [4].

*KMT2A* rearrangements (*KMT2A*-r) are among the most common recurrent genetic aberrations in pediatric AML, occurring in 24% of newly diagnosed patients [2]. To date, more than 90 different fusion partner genes of *KMT2A* have been identified. However, in the setting of pediatric and infant AML, *KMT2A/MLLT3* and *KMT2A/MLLT10* translocations are among the most frequent *KMT2A*-r [5]. The most common fusion partner genes of *KMT2A* are functionally related and interact in a protein complex [6,7]. *KMT2A* has histone H3-lysine-4-methyltransferase activity, and *KMT2A* fusion proteins recruit the super elongation complex, thereby causing transcriptional elongation [8,9]. As a result, *KMT2A*-r effects leukemogenesis, for example, by inducing high HOXA gene expression [10,11]. 

The outcome of patients with *KMT2A*-rearranged AML is dependent on the fusion partner genes [12,13]. Approximately a decade ago, Balgobind et al. studied the outcome of pediatric AML patients with different *KMT2A*-r in a large, multicentric analyzed cohort [12]. It was shown that patients with *KMT2A/AFDN* translocations had the worst outcomes. Translocation of *KMT2A/MLLT10* also showed a poor prognosis, whereas patients with *KMT2A/MLLT11* translocations showed an event-free survival rate of 92% and an overall survival rate of 100%. With an overall survival of 63%, this study showed a favorable prognosis for the most frequent *KMT2A* fusion partner gene, *MLLT3* [12]. The same trends were confirmed in an adult AML cohort [13].

The number of additional aberrations in AML with *KMT2A*-r is low compared to patients without these aberrations [10,13]. However, genes of the RAS pathway, such as *KRAS*, *NRAS* and *PTPN11*, are commonly mutated in AML with *KMT2A*-r [10,13,14]. 

CSPG4, previously named human homologue of NG2, is absent on the surface of healthy cells of the hematopoietic system, but is aberrantly expressed on the leukemic blasts of a proportion of pediatric (11–35%) and adult AML (13–36%) patients, as well as in ALL [15,16,17,18,19]. The expression level of CSPG4 is heterogeneous between patients, and CSPG4 expression was shown to be strongly associated with *KMT2A*-r [15,16,19]. Although CSPG4 is frequently expressed on the surface of AML cases with *KMT2A*-r, it is not expressed on the blasts of every AML patient with *KMT2A*-r. In AML, CSPG4 expression is not dependent on specific fusion partner genes of *KMT2A*. Both *KMT2A*-r and CSPG4 expression correlate with the monocytic morphology of blasts [15,20]. 

To date, most analyzes regarding the impact of *KMT2A*-r on outcomes in AML have been multicentric, including various treatment protocols [12,21]. Here, we present the clinical, genetic and multicolor flow cytometric data of 967 pediatric AML patients who were enrolled onto one of the German pediatric AML-BFM studies and registries between 2004 and 2019. Since 97% of all pediatric AML patients who develop leukemia in Germany are enrolled onto one of the AML-BFM studies [3], this cohort is population-based which reflects the German population. Patients were enrolled onto the trials AML04 and AMLS12 and the registries AMLR12 and AMLR17, all of which had comparable treatment protocols. Therefore, we present a large population-based cohort of equally treated pediatric AML patients. We compared the influence of different *KMT2A*-r on outcomes of treatment and analyzed the presence of additional cytogenetic and molecular genetic aberrations, as well as the co-expression of the surface molecule CSPG4. 

## 2. Materials and Methods

### 2.1. Study Cohort

We investigated 967 pediatric AML patients (<18 years) with diagnosis until 2019 (excluding FAB M3 and Down Syndrome) and known *KMT2A*-r status. All analyzed patients were treated in Germany and enrolled onto one of the following AML-BFM trials or registries: AML-BFM 04 trial (ClinicalTrials.gov Identifier: NCT00111345), AML-BFM 2012 trial or registry (EudraCT number: 2013-000018-39) and AML-BFM 2017 registry (DRKS number: DRKS00013030). All trials and registries were approved by the ethical committees and institutional review boards of the university hospitals of Münster, Hannover and Essen in accordance with the declaration of Helsinki. For patients taking part in all trials and registries, written informed consent was obtained before beginning treatment. Treatment protocols of all analyzed trials and registries include similar cytarabine-/daunorubicin-based induction chemotherapy. Duration of maintenance treatment and indication of stem cell transplantation in each trial was dependent on the risk group stratification. Standard procedures for diagnostics, especially for morphologic, immune phenotypic and molecular genetic diagnostics, were carried out in the German AML-BFM reference laboratory, except the classical cytogenetic analysis which was performed in the institute for human genetics in Hannover. As 97% of all German patients diagnosed with pediatric AML are enrolled onto one of the AML-BFM trials, our cohort represents the German population [3].

### 2.2. Genetic Data

Depending on the original trial and time of diagnosis, the *KMT2A*-r analysis was performed in bone marrow or peripheral blood samples using the following methods: classical karyotyping, fluorescence in situ hybridization or multiplex polymerase chain reaction [22]. In addition, RNA sequencing was carried out in 105 of the 967 patients. RNA sequencing was performed using the TruSight RNA Fusion Panel (Illumina, San Diego, CA, USA) following the manufacturer’s recommendations. Sequencing was performed on an Illumina MiSeqDX sequencer in research mode with 76 bp paired-end reads using the MiSeq Reagent Kit v3 (150-cycle). For data analysis, including fusion calling, the RNA Fusion Analysis Module v2.0 of the local run manager of the MiSeqDX system was used. Additional genetic aberrations were identified by fragment length analysis for the detection of *FLT3* internal tandem duplications (*FLT3*-ITD) and mutation screening with the TruSight Myeloid Panel (Illumina, San Diego, CA, USA) (309/967). The sample preparation for mutational screening was prepared according to the manufacturer’s instructions. Sequencing was performed on an Illumina MiSeqDX sequencer in research mode with 156 bp paired-end reads using the MiSeq Reagent Kit v2 (300-cycle). For data analysis, SOPHiA DDM™ Software was used. Classical karyotyping and fluorescence in situ hybridization were performed in the institute for human genetics in Hannover. The subcohort of patients that was analyzed with mutational screening comprised all patients with diagnosis of AML since 2016 and some randomly selected cases that were retrospectively analyzed (Table S1).

### 2.3. Flow Cytometric Data

Between 2010 and 2018, a subcohort of 346 patients was analyzed for CSPG4 expression via flow cytometry (Supplementary Table S2). The PE conjugated antibody NG2 (clone 7.1) from Beckman Coulter was used for the detection of CSPG4 expression on AML blasts. Leukemic cells were identified as recommended [22]. CSPG4 expression levels were determined and grouped according to the AIEOP-BFM consensus guidelines for antigen expression rating [23].

### 2.4. Statistical Analysis

The Kaplan–Meier method was used to determine the probability of overall (OS) and event-free survival (EFS). As we expect to notice every event of each patient with pediatric AML in Germany, patients without any events were censored at the time of the analysis for both the OS and EFS. OS (or EFS) was determined in years from the diagnosis to death (or first event) or until this analysis. Events were defined as relapse, death, secondary malignancy, non-response to treatment (no complete remission after second induction) as well as early death (<43 days after diagnosis). Early death, as well as non-response, were defined as events at diagnosis. Survival curves were compared using the log-rank test. All other statistical calculations were carried out using either an unpaired *t*-test or Pearson’s Chi-Square test. Differences with a *p*-value less than 0.05 were considered statistically significant. All statistical analyzes were performed using IBM^®^ SPSS Statistics, version 27. For the generation of diagrams, GraphPad Prism, version 6, was used. 

## 3. Results

### 3.1. Patient Characteristics

**Figure 1:**
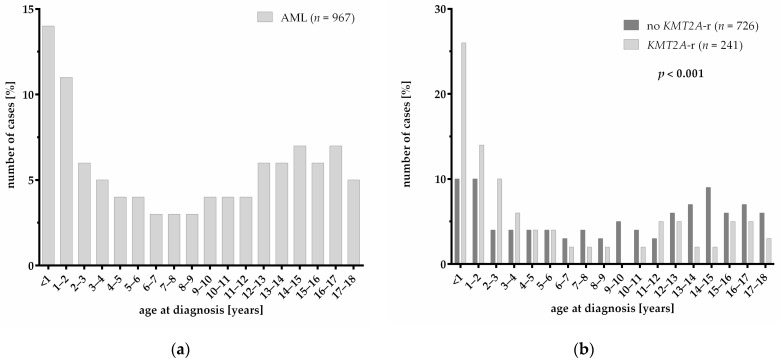
Age distribution of pediatric AML patients: (**a**) Age distribution of the total cohort of pediatric AML patients. (**b**) Comparison of age distribution of pediatric AML patients with and without *KMT2A*-r (*p* < 0.001). Significance was calculated with Pearson’s chi square test.

Our cohort of 967 pediatric AML patients includes 241 patients with *KMT2A*-r (24.9%) as shown by classical karyotyping, fluorescence in situ hybridization, multiplex PCR or targeted RNA sequencing and 726 patients without *KMT2A*-r (75.1%) (Table 1). Patients with *KMT2A*-r were significantly younger at diagnosis with a median age of 3.3 years, compared to patients who were not carrying *KMT2A*-r with a median age of 10.0 years (*p* < 0.001). The age distribution of pediatric AML patients (Figure 1a) divides into two main peaks in infancy (<12 months) and adolescence (14–17 years). However, most of the patients with *KMT2A*-r develop leukemia as an infant (<12 months) or at an early childhood age (1–3 years) (Figure 1b). Patients without *KMT2A*-r show a more even age distribution (*p* < 0.001). Gender distribution did not differ significantly between the two groups. In patients with *KMT2A*-r, the white blood cell (WBC) count (*p* = 0.001) and platelet count (*p* < 0.001) at diagnosis were significantly increased compared to patients without *KMT2A*-r (Table 1). Regarding the morphology of AML blasts, patients with *KMT2A*-r showed a significant increase in the monocytic morphology—the FAB subtype M5 occurred particularly frequently (*p* < 0.001). Distribution into risk groups was based on the molecular genetics and response to therapy. With respect to previous observations, *KMT2A*-r strongly influences the stratification into risk groups. The distribution of risk groups therefore significantly differs between patients with and without *KMT2A*-r (*p* < 0.001) with greatly increased proportions of patients with intermediate and high risk in the group of patients carrying *KMT2A*-r.

### 3.2. KMT2A-r and Subgroups

A more detailed analysis of the 241 patients with *KMT2A*-r identified 13 different *KMT2A* fusion partner genes and a group for which the fusion partner gene could not be identified. The latter is, therefore, named *KMT2A-r not further specified* (Table 2). The most frequent types of *KMT2A*-r were *KMT2A/MLLT3* (43%, *n* = 104) and *KMT2A/MLLT10* (23%, *n* = 55) fusions, followed by *KMT2A/AFDN* (5%, *n* = 11) and *KMT2A/MLLT1* (5%, *n* = 11). Because of the small number of patients, all other *KMT2A* subgroups were grouped as *KMT2A other* in the analysis of survival curves. *KMT2A* subgroups and survival data are described in more detail in Table 2.

### 3.3. Genetics

**Figure 2:**
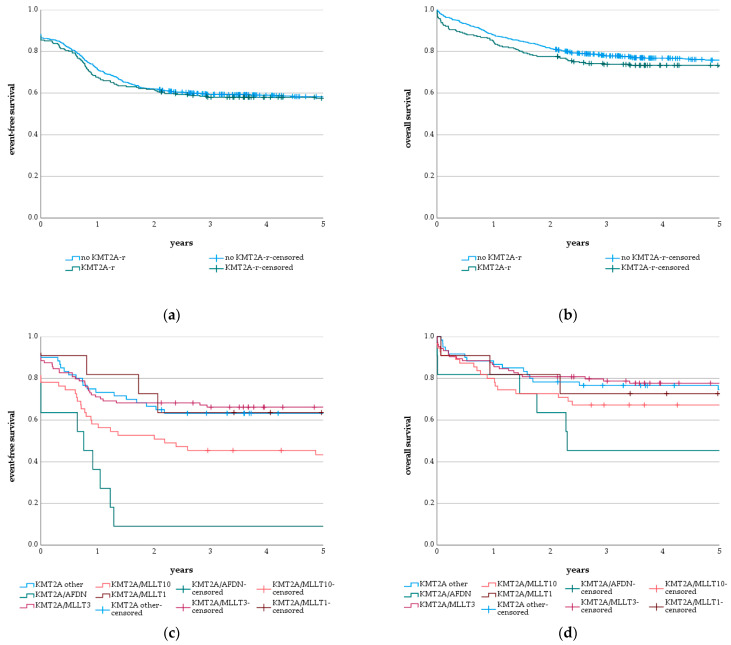
Survival of pediatric AML patients depending on *KMT2A* rearrangements (*KMT2A*-r) and subgroups. (**a**) 5-year event-free survival (EFS) of pediatric AML patients with *KMT2A*-r (57.5 ± 3.2%) and without *KMT2A*-r (58.3 ± 1.8%) (*p* = 0.640); (**b**) 5-year overall survival (OS) of pediatric AML patients with *KMT2A*-r (72.8 ± 2.9%) and without *KMT2A*-r (75.8 ± 1.6%) (*p* = 0.298); (**c**) 5-year event-free survival (EFS) of pediatric AML patients with the translocations *KMT2A/AFDN* (9.1 ± 8.7%), *KMT2A/MLLT1* (63.6 ± 14.5%), *KMT2A/MLLT3* (66.3 ± 4.6%), *KMT2A/MLLT10* (43.4 ± 6.7%) or other *KMT2A*-r (63.3 ± 6.2%) (*p* < 0.001); (**d**) 5-year overall survival (OS) of pediatric AML patients with the translocations *KMT2A/AFDN* (45.5 ± 15.0%), *KMT2A/MLLT1* (72.7 ± 13.4%), *KMT2A/MLLT3* (77.7 ± 4.1%), *KMT2A/MLLT10* (67.3 ± 6.3%) or other *KMT2A*-r (74.6 ± 5.7%) (*p* = 0.176). Significance was calculated with log-rank test.

In order to analyze the impact of *KMT2A*-r on the outcome of pediatric AML patients, the overall survival (OS) and event-free survival (EFS) of the 241 patients with (OS = 72.8 ± 2.9%; EFS = 57.5 ± 3.2%) and the 726 patients without *KMT2A*-r (OS = 75.8 ± 1.6%; EFS = 58.3 ± 1.8%) were compared. This comparison did not show any significant difference between both groups, neither for OS (*p* = 0.298; Figure 2b) nor EFS (*p* = 0.640; Figure 2a). Separately analyzing the impact of *KMT2A*-r in patients with standard and intermediate risk or high risk also did not show a significant difference between OS and EFS (Supplementary Figure S1). However, the ratio of early death to other cases of death was significantly higher in AML with *KMT2A*-r (ratio: 0.38) compared to patients without *KMT2A*-r (ratio: 0.13) (*p* = 0.002). Particularly, the most frequent rearrangements showed high ratios of early death to other cases of death, indicating a severe therapy-independent impact on overall survival: *KMT2A/MLLT3* (ratio: 0.44), *KMT2A/MLLT10* (ratio: 0.38), *KMT2A/AFDN* (ratio: 0.50) and *KMT2A/MLLT1* (ratio: 0.50). Thereby, the WBC count of cases with *KMT2A*-r and early death was increased accompanied by a higher occurrence of hyperleukocytosis compared to patients with *KMT2A*-r but without early death. However, the WBC count of cases with early death but without *KMT2A*-r was also increased and hyperleukocytosis occurred more frequently in this group. The infrequent types of *KMT2A*-r, *KMT2A/SEPTIN9* (*n* = 3) and *KMT2A/MLLT6* (*n* = 2), did not show a single case of death during a median follow up of 7.0 and 10.6 years, respectively. Additionally, single patients with rare translocations *KMT2A/ABI2* (*n* = 1; follow up: 2.6), *KMT2A/USP2* (*n* = 1; follow up: 2.1) and *KMT2A/KNL1* (*n* = 1; follow up: 6.0) remained event-free during the indicated observation times. Even though the OS of patients with *KMT2A*-r subgroups did not differ significantly (*p* = 0.176), there was a significant difference between the event-free survival of the different types of *KMT2A*-r (*p* < 0.001). *KMT2A/AFDN* translocations showed the lowest probability of 5-year EFS of 9.1 ± 8.7%, followed by patients with *KMT2A/MLLT10* translocations with a 5-year EFS of 43.4 ± 6.7%. *KMT2A/MLLT3* (OS = 77.7 ± 4.1%; EFS = 66.3 ± 4.6%); *KMT2A/MLLT1* translocations (OS = 72.7 ± 13.4%; EFS = 63.6 ± 14.5%), together with the group of *KMT2A other* (OS = 74.6 ± 5.7%; EFS = 63.3 ± 6.2%), exhibited the best OS and EFS (Figure 2c,d).

**Figure 3:**
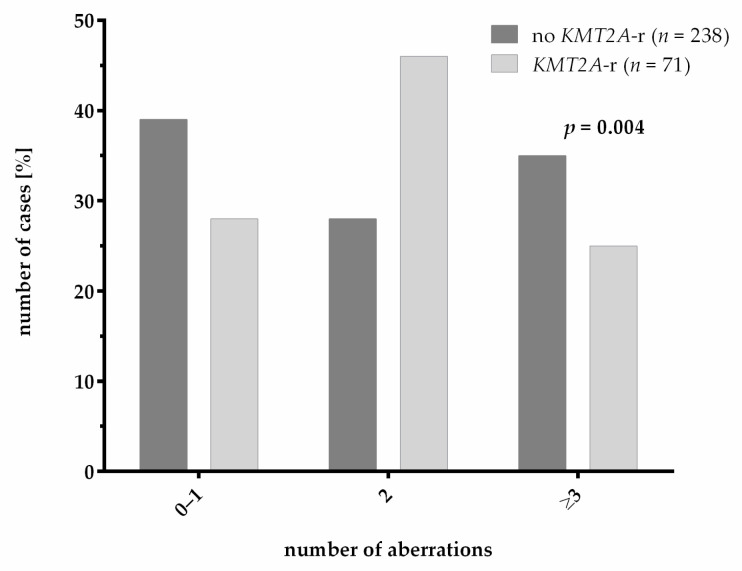
Number of genetic aberrations in pediatric AML patients with and without *KMT2A*-r (*p* = 0.004). Significance was calculated with Pearson’s chi square test.

For the analysis of co-occurrence of additional cytogenetic and molecular genetic aberrations, we analyzed a total of 309 patients that were at least tested with classical cytogenetics and mutational screening with the use of targeted next-generation sequencing. A comparison of the clinical characteristics in this subcohort with the cohort that was not analyzed with panel sequencing revealed a significant bias in age and WBC count, presenting the clinical reality of our studied cohort, which should not have had an effect on the subsequent analyses of the co-occurrence of additional aberrations (Table S1). The additional bias in the distribution of risk groups is probably due to the overall improvements in the detection of variants (fusions + mutations) by next-generation sequencing methods, leading to improved risk group stratification. We found a significant difference in the number of genetic aberrations occurring in patients with and without *KMT2A*-r (*p* = 0.004; Figure 3). In AML with *KMT2A*-r, the majority of patients showed only one additional aberration, having a total of two aberrations. In contrast, most patients without *KMT2A*-r harbored zero to one or more than two aberrations.

We observed a significant positive correlation between *KMT2A*-r and mutations in the *KRAS* gene (*p* < 0.001). However, no impact of *KRAS* mutations on the outcome of patients with *KMT2A*-r could be observed (Figure S2). Interestingly, no *KRAS* mutation was detected in patients with *KMT2A/MLLT1* translocation (*n* = 6) (Table S3).

Other genetic aberrations that occurred frequently in AML with *KMT2A*-r are trisomy 8, *NRAS* mutations and *FLT3*-TKD mutations. However, none showed a significant co-occurrence. *FLT3*-ITDs were found significantly less frequently in AML with *KMT2A*-r (*p* < 0.001). Additionally, *KMT2A*-r were mutually exclusive with mutations in *NPM1* (*p* = 0.002), *KIT* (*p* = 0.036), *WT1* (*p* < 0.001), *CEBPA* (*p* = 0.006) and the translocations *NUP98/NSD1* (*p* = 0.009), *RUNX1/RUNX1T1* (*p* = 0.003) and *CBFB/MYH11* (*p* = 0.006), considering only translocations occurring recurrently in at least 10 patients without *KMT2A*-r (Table S4).

### 3.4. CSPG4 Expression

**Figure 4:**
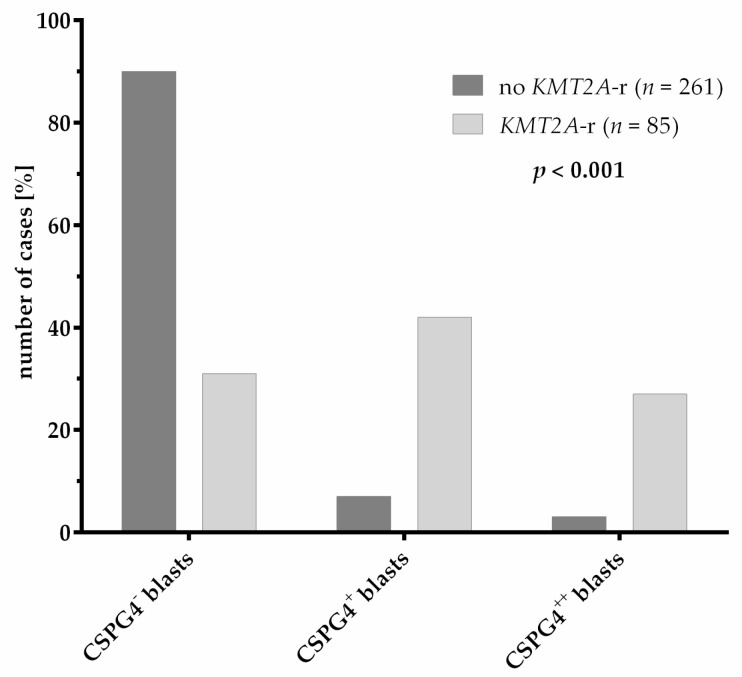
CSPG4 expression levels on blasts of pediatric AML patients with and without *KMT2A*-r (*p* < 0.001). Significance was calculated with Pearson’s chi square test. CSPG4^−^ = no CSPG4 expression (<10% of blasts); CSPG4^+^ = weak CSPG4 expression (10–50% of blasts); CSPG4^++^ = strong CSPG4 expression (>50% of blasts).

Eighty-five patients with and 261 patients without *KMT2A*-r were analyzed for surface expression of CSPG4 on AML blasts at diagnosis. A comparison of clinical characteristics between the patients analyzed and not analyzed for CSPG4 expression showed a discrepancy in the distribution of risk groups (Table S2). This is probably caused by the continuous improvement of the detection of variants (fusions and mutations), which guides the risk group stratification, during the collection of cases for the CSPG4 expression analysis. The analysis showed that *KMT2A*-r significantly correlates with CSPG4 expression on the surface of AML blasts (*p* < 0.001) (Table 1). However, not all patients with *KMT2A*-r showed detectable CSPG4 expression and the expression of CSPG4 was also found on blasts of AML patients without *KMT2A*-r (Figure 4). Even a strong expression, defined by the detection of CSPG4 on >50% of blasts, was not indicative of *KMT2A*-r. To elucidate the mechanism causing the aberrant strong (>50% of blasts, *n* = 8) and weak (10–50% of blasts, *n* = 19) expression of CSPG4 in cases without *KMT2A*-r, we analyzed the genetic data of these cases. Interestingly, 3/19 patients with weak CSPG4 expression and 2/8 patients with strong CSPG4 expression showed genetic aberrations in other epigenetic regulators (Table S5). Thus, we were able to identify single cases with weak CSPG4 expression carrying the translocations *KAT6A/NCOA2* (1/19) and *NUP98*/*KDM5A* (1/19), and one case displaying a *KDM6A* mutation (1/19) without having *KMT2A*-r. Additionally, the translocations *KAT6A/EP300* (1/8) and *KAT6A/CREBBP* (1/8) were detected in the cases with strong CSPG4 expression but without *KMT2A*-r. Except from aberrations in epigenetic regulators, *CBFB/MYH11* translocations (10/19) and *WT1* mutations (3/19) were frequent in weak CSPG4-positive cases.

**Figure 5:**
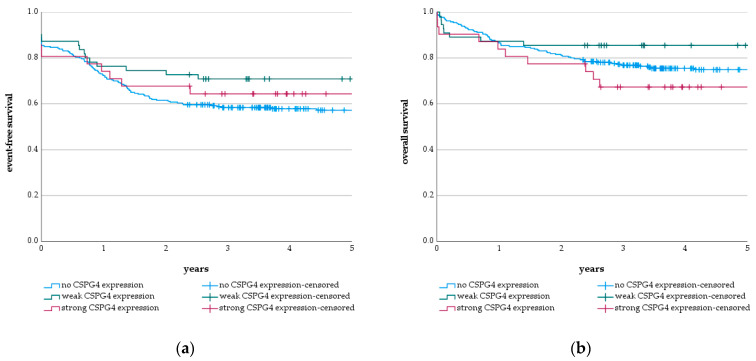
Survival of pediatric AML patients depending on CSPG4 expression level. (**a**) 5-year event-free survival (EFS) of pediatric AML patients without CSPG4 expression (57.2 ± 3.1%), with weak CSPG4 expression level (70.9 ± 6.1%) and with strong CSPG4 expression level (64.4 ± 8.6%) (*p* = 0.204); (**b**) 5-year overall survival (OS) of pediatric AML patients without CSPG4 expression (74.9 ± 2.7%), with weak CSPG4 expression level (85.5 ± 4.8%) and with strong CSPG4 expression level (67.3 ± 8.5%) (*p* = 0.161). Significance was calculated with log-rank test.

Analysis of the impact of CSPG4 expression levels as a prognostic factor showed neither a significant effect on OS (Figure 5a; *p* = 0.161) nor on the EFS (Figure 5b; *p* = 0.204) of the pediatric AML patients. In addition, CSPG4 expression did not have a prognostic significance in AML with *KMT2A*-r (OS: *p* = 0.398, EFS: *p* = 0.526) (Figure S3). Thus, a strong association for CSPG4 expression on the blasts of pediatric AML with *KMT2A*-r was identified. However, this CSPG4 expression had no prognostic significance.

## 4. Discussion

In this work, we present the results of our comparison of a German population-based cohort of pediatric AML patients with and without *KMT2A*-r. The parameters analyzed were clinical characteristics, treatment outcome, co-occurrence of additional genetic aberrations and the expression of CSPG4 on the cell surface of AML blasts.

In accordance with previous studies, we showed that pediatric AML patients with *KMT2A*-r had a younger age and higher WBC count at diagnosis compared to patients without *KMT2A*-r [20,24]. Regarding the age, we observed that *KMT2A*-r mostly occur between the ages 0 to 3 years and are mainly responsible for the two-peak age distribution of pediatric AML. As previously described, *KMT2A*-r was strongly associated with monocytic morphology, especially with the FAB subtype M5 [20]. As risk group stratification is strongly based on molecular genetics as well as on therapy response, it was not surprising that the distribution of risk groups correlated with the distribution of *KMT2A* fusion partner genes [25]. Nevertheless, some differences in risk group stratification and distribution of *KMT2A* subgroups are due to the availability of newer methods with higher sensitivity (TruSight RNA Fusion Panel) which allowed the retrospective identification of *KMT2A* translocation partner genes, which would have resulted in a different risk group stratification and, thus, different treatment. Despite the differences in distribution of risks groups, *KMT2A*-r had no impact on EFS and OS. This underlines the efficiency of the risk-adapted treatment approach of the recent protocols, showing remarkable survival rates for patients with high-risk AML mainly resulting from the inclusion of hematopoietic stem cell transplantations (HSCT) as the standard treatment for a high-risk patient accompanied by improved HSCT protocols with less morbidity and mortality over the years [26,27].

In our cohort of 241 pediatric AML patients with *KMT2A*-r, we identified 13 different translocation partner genes. In line with previous reports, *KMT2A/MLLT3* (43%) was the most frequent subgroup of *KMT2A*-r, followed by *KMT2A/MLLT10* (23%), *KMT2A/AFDN* (5%) and *KMT2A/MLLT1* (5%) [5]. *KMT2A/ELL* translocations were less frequently observed in our cohort than in the study of Meyer et al. [5]. In accordance with Balgobind et al., we observed a translocation-partner-gene-dependent difference in the survival outcome of *KMT2A*-r patients [12]. Compared to the analysis of Balgobind et al. in 2009, we showed improved overall survival rates of all four subgroups of *KMT2A*-r in our study [12]. We also observed a better event-free survival compared to the analysis of Balgobind et al. Only patients with *KMT2A/AFDN* translocations also showed a poor event-free survival in our analysis [12]. However, the patient cohort of Balgobind et al. was a multicentric study collecting data from many different countries with different treatment protocols, whereas our study only included patients from Germany receiving similar treatment over many years. Due to the rapid development of a new, more sensitive methodology, especially for molecular genetic diagnostics, risk stratification and, therefore, risk-adapted treatment has been widely improved since the analysis of Balgobind et al. A further major difference between the cohort of Balgobind et al. and our analyzed cohort is the inclusion of HSCT as part of the treatment protocol for high-risk patients. In this regard, Klusmann et al. already showed better survival rates for patients with *KMT2A*-r allocated to HSCT, which was further confirmed in the study of Rasche et al. [4,26,27]. Here, we were able to discriminate between *KMT2A*-r, which benefits from stratification into a high-risk group (*KMT2A/AFDN*; *KMT2A/MLLT10*; *KMT2A/AFF1*), and those who could be cured with chemotherapy. Therefore, accurate molecular diagnostics at the beginning of the disease is very important to allow risk stratification to be performed correctly; thus, the appropriate treatment protocol can be selected for each patient. Our analysis highlighted that early deaths were more frequent in AML with *KMT2A*-r compared to non-*KMT2A*-rearranged AML. This was accompanied by a higher WBC count and higher occurrence of hyperleukocytosis. This was also seen in patients with early death but without *KMT2A*-r. The treatment protocols of this study already include the treatment of patients with high WBC count with hydroxyurea, exchange transfusion or leukapheresis, as the benefit of this treatment was shown by Creutzig et al. in 2015 [28]. Nevertheless, our data suggest that the applied treatment options are insufficient for patients with co-occurrence of high WBC and *KMT2A*-r and need further adjustments. The underlying mechanism for the higher early death ratio in patients with *KMT2A*-r compared to patients without *KMT2A*-r remains elusive and needs additional investigation.

AML with *KMT2A*-r was previously described to have fewer numbers of genetic aberrations compared to AML with wildtype *KMT2A* [10,13]. In our analysis, AML with *KMT2A*-r harbored additional mutations in approximately 70% of cases. However, in accordance with Bill et al., we show that patients with *KMT2A*-r mostly harbored only one additional aberration whereas patients without *KMT2A*-r more frequently harbored three or more aberrations [13]. Our finding that *KRAS* was frequently mutated in *KMT2A*-r pediatric AML is in line with other studies [10,13,14,29]. Matsuo et al. observed a significant adverse impact of *KRAS* mutations on the survival of patients with *KMT2A*-r [29]. However, we did not observe a difference in the outcome of *KMT2A*-r patients with or without *KRAS* mutation in our study. Interestingly, previous studies observed a correlation of the occurrence of *KMT2A/AFDN* translocations with *KRAS* mutations and higher numbers of additional aberrations. Both were described as adverse prognostic factors, possibly resulting in the severe prognosis that could be observed in our study and in previous studies [29,30]. Unfortunately, in our study, the case number carrying these aberrations was too low (*n* = 3) for an analysis addressing this question (Table S3). *NRAS* and *FLT3*-TKD mutations frequently occurred in AML with *KMT2A*-r, but without statistical significance. This observation was in line with previous studies that identified genes of the RAS-pathway as frequently mutated in AML with *KMT2A*-r [10,13,14]. In our cohort, *FLT3*-ITDs occurred less frequently in AML with *KMT2A*-r. This was in accordance with the observations of Bill et al. [13]. Although trisomy 8 occurred frequently in AML with *KMT2A*-r in our cohort, we were not able to detect a significant correlation of *KMT2A*-r and trisomy 8 and the associated significant favorable impact on the survival of these patients due to reduced relapse rates as described by Coenen et al. [30]. In line with previous observations, we found *KMT2A*-r to be mutually exclusive with *RUNX1/RUNX1T1*, *NUP98/NSD1* and *CBFB/MYH11* translocations as well as with *NPM1*, *KIT*, *WT1* and *CEBPA* mutations [10]. In contrast, in adult AML with *KMT2A*-r, Bill et al. described infrequent mutations of *NPM1* and *CEBPA*, underlining the differences between pediatric and adult AML [13].

Surface molecule CSPG4 is not expressed on healthy hematopoietic cells [15]. As the association of *KMT2A*-r with CSPG4 expression is widely known, its expression is used in diagnostics as the first indicator of *KMT2A*-r. In the past, CSPG4 was thought to be exclusively expressed on blasts with *KMT2A*-r [15,16]. Here, we show that the outcome of patients with *KMT2A*-r is independent from the CSPG4 expression level and that the CSPG4 expression is not exclusive for cases with *KMT2A*-r independent from the CSPG4 expression level. Thus, CSPG4 cannot be used as a surrogate marker for *KMT2A*-r in diagnostics. We also observed CSPG4 expression on blasts of patients with point mutations or translocations of other epigenetic regulators such as lysine acetyltransferases or lysine demethylases. The exact mechanism behind the upregulation of CSPG4 expression in AML with *KMT2A*-r is yet to be determined; nevertheless, some of the aberrations in epigenetic regulators that were found in CSPG4-expressing blasts without *KMT2A*-r are described to show similar *HOXA* gene expression profiles to *KMT2A*-r [31,32]. As wildtype KMT2A and KAT6A interact to activate HOXA9, a similar regulation of CSPG4 expression might be possible [33]. Hence, the question arises, can similar pathways for the regulation of CSPG4 expression be activated? Surprisingly, weak CSPG4 expression with missing *KMT2A*-r strongly correlated with good prognostic translocation of *CBFB/MYH11*. In addition, the blasts of cases with *CBFB/MYH11* showed significantly higher expression levels of *HOXB* cluster genes and *MEIS1* compared to blasts of cases with other favorable aberrations, such as *RUNX1/RUNX1T1* [34]. As MEIS1 upregulation seems to be important in AML with *KMT2A*-r as well as *CBFB/MYH11*, it is of interest to investigate the downstream regulation of these translocations and the link to the upregulation of CSPG4 further [34,35].

## 5. Conclusions

In summary, in this German population-based study, we have examined the impact of *KMT2A*-r on the clinical characteristics and outcome of pediatric AML patients, as well as the correlation of *KMT2A*-r with additional genetic aberrations and the expression of CSPG4 on the cell surface.

Although *KMT2A*-r associated with the FAB subtype M5, high white blood cell count and younger age at diagnosis, the *KMT2A*-r status did not influence the OS and EFS. However, a detailed analysis of the *KMT2A*-r subgroups revealed clear differences in the prognosis of the disease, and also highlighted the importance of refined risk stratification groups according to genetic alterations and the successful implementation of HSCT as standard of care for high risk patients. This underlines the importance of accurate molecular diagnostics to successfully identify the different *KMT2A*-r subgroups to be able to apply the correct treatment protocol for each patient. In addition, the co-occurrence of *KMT2A*-r with additional cytogenetic and molecular genetic aberrations further highlighted the heterogeneity of AML with *KMT2A*-r. Future analysis should address this heterogeneity and its impact on the prognosis of pediatric AML in bigger cohorts, elucidating the underlying mechanisms causing these differences. In this regard, the aberrant expression of CSPG4 and its connection with *KMT2A*-r, other epigenetic regulators and *CBFB/MYH11* translocation need further investigation.

## Figures and Tables

**Table 1:** Clinical characteristics of pediatric AML patients with and without *KMT2A*-r at diagnosis.

Features		All Patients	Patients with *KMT2A*-r	Patients without *KMT2A*-r	*p*-Value
Number		967 (100%)	241 (25%)	726 (75%)	-
Age	Median	8.3	3.3	10.0	<0.001 ^T^ (***)
	Range	0.0–18.0	0.0–17.9	0.0–18.0	
Gender	Male	491 (100%)	134 (27%)	357 (73%)	0.084 ^C^ (n.s.)
	Female	476 (100%)	107 (22%)	369 (78%)	
WBC count [×10^3^/µL]	Median	16.1	19.3	15.8	0.001 ^T^ (**)
	Range	0.0–817.1	0.2–585.0	0.0–817.1	
Hemoglobin [g/dL]	Median	8.3	8.3	8.3	0.156 ^T^ (n.s.)
	Range	2.1–18.3	2.1–17.1	3.1–18.3	
Platelet count [×10^3^/µL]	Median	66.0	89.0	60.0	<0.001 ^T^ (***)
	Range	2.0–1370.0	5.0–468.0	2.0–1370.0	
Risk groups	Standard risk	244 (27%)	4 (2%)	240 (35%)	<0.001 ^C^ (***)
	Intermediate risk	552 (61%)	185 (81%)	367 (54%)	
	High risk	114 (13%)	40 (17%)	74 (11%)	
	No data	57	12	45	-
Morphologic subtype (FAB classification)	M0	19 (2%)	2 (1%)	17 (%)	<0.001 ^C^ (***)
	M1	112 (14%)	6 (3%)	106 (18%)	
	M2	175 (22%)	4 (2%)	171 (28%)	
	M4	134 (17%)	36 (18%)	98 (16%)	
	M4 eo	67 (8%)	0 (0%)	67 (11%)	
	M5	198 (25%)	147 (73%)	51 (8%)	
	M6	12 (1%)	1 (0%)	11 (2%)	
	M7	88 (11%)	6 (3%)	82 (14%)	
	No data	162	39	123	-
CSPG4 expression	Strong positive	31 (9%)	23 (27%)	8 (3%)	<0.001 ^C^ (***)
	Weak positive	55 (16%)	36 (42%)	19 (7%)	
	Negative	260 (75%)	26 (31%)	234 (90%)	
	No data	621	156	465	-

^T^ unpaired *t*-test; ^C^ Pearson’s chi square test; ** *p* < 0.01; *** *p* < 0.001; n.s. not significant.

**Table 2:** Outcome of Pediatric AML Patients with Specific *KMT2A*-r.

Features	Total	Alive	Relapsed	Dead (Early Death)	Non- Response	Secondary Malignancy
no *KMT2A*-r	726	547	206	179 (20)	97	6
*KMT2A/MLLT3*	104	81	20	23 (7)	12	1
*KMT2A/MLLT10*	55	37	16	18 (5)	12	2
*KMT2A/AFDN*	11	5	7	6 (2)	4	0
*KMT2A/MLLT1*	11	8	3	3 (1)	1	0
*KMT2A/MLLT11*	8	7	1	1 (1)	1	0
*KMT2A/AFF1*	3	2	1	1 (0)	0	0
*KMT2A/SEPTIN9*	3	3	0	0 (0)	0	0
*KMT2A/ELL*	3	2	1	1 (0)	0	0
*KMT2A/MLLT6*	2	2	0	0 (0)	0	0
*KMT2A/PRPF19*	2	1	0	1 (0)	0	0
*KMT2A/ABI2*	1	1	0	0 (0)	0	0
*KMT2A/KNL1*	1	1	1	0 (0)	0	0
*KMT2A/USP2*	1	1	0	0 (0)	0	0
*KMT2A*-r not further specified	36	24	10	12 (2)	4	0

## Data Availability

A detailed data sharing statement is provided in the Supplementary Materials.

## Supplementary Materials

The following are available online at https://www.mdpi.com/article/10.3390/cancers13194817/s1, Figure S1: Survival of pediatric AML patients depending on *KMT2A* rearrangements (*KMT2A*-r) and risk group; Figure S2: Survival of pediatric AML patients with *KMT2A*-r and with or without *KRAS* mutation; Figure S3: Survival of pediatric AML patients with *KMT2A*-r depending on CSPG4 expression level; Table S1: Clinical characteristics of pediatric AML patients with and without mutational screening via next-generation sequencing using the Illumina TruSight Myeloid Panel (TSM); Table S2: Clinical characteristics of pediatric AML patients with and without analysis for CSPG4 expression via multicolor flow cytometry; Table S3: *KRAS* mutations and correlation with specific *KMT2A*-r; Table S4: Genetic aberrations and dependency on *KMT2A*-r status; Table S5: Samples of patients with weak or strong CSPG4 expression and associated gene fusions and mutations.
