# Supplementary material for: Impact of KMT2A Rearrangement and CSPG4 Expression in Pediatric Acute Myeloid Leukemia
Source: Cancers (Basel). 2021 Sep 26;13(19):4817. doi: 10.3390/cancers13194817 (PMC8508499; doi:10.3390/cancers13194817)
Supplement: Supplementary file 1 [file cancers-13-04817-s001.zip › cancers-1401710-supplementary.pdf]

cancers

MDPI

Article

Impact of KMT2A Rearrangement and CSPG4 Expression in Pediatric Acute Myeloid Leukemia

Lina Marie Hoffmeister [1,*], Eser Orhan [2], Christiane Walter [1], Naghmeh Niktoreh [1], Helmut Hanenberg [1,3], Nils von Neuhoff [1], Dirk Reinhardt [1] and Markus Schneider [1,*]

Supplementary Material

1. Data Sharing Statements BFM

The AML-BFM Study Group Data Sharing policy describes the release and use of AML-BFM individual subject data for use in research projects in accordance with EU-Directive of Good Clinical Practice, the guidelines of the German Research Foundation (DFG) and the German Society of Pediatric Oncology and Hematology (GPOH). Only data expressly released from the oversight of the relevant AML-BFM Data and Safety Monitoring Committee (DSMC) are available to be shared. Data sharing will ordinarily be considered only after the primary study manuscript is accepted for publication. For phase III trials, individual-level de-identified datasets that would be sufficient to reproduce results provided in a publication containing the primary study analysis can be requested from the AML-BFM data management. Data are available to researchers who wish to analyze the data in secondary studies to enhance the public health benefit of the original work and agree to the terms and conditions of use. For non-phase III trials, data are available following the primary publication. An individual-level de-identified dataset containing the variables analyzed in the primary results paper can be expected to be available upon request. Requests for access to AML-BFM protocol research data should be sent to the AML-BFM Study Group offices. Data are available to researchers whose proposed analysis is found by the AML-BFM research board to be feasible and of scientific merit and who agree to the terms and conditions of use. For all requests, no other study documents, including the protocol, will be made available and no end date exists for requests. In addition to above, release of data collected in a clinical trial conducted under a binding collaborative agreement between AML-BFM Study Group and a pharmaceutical/biotechnology company must comply with the data sharing terms of the binding collaborative/contractual agreement and must receive the proper approvals.

2. Supplementary Figures S1–S3

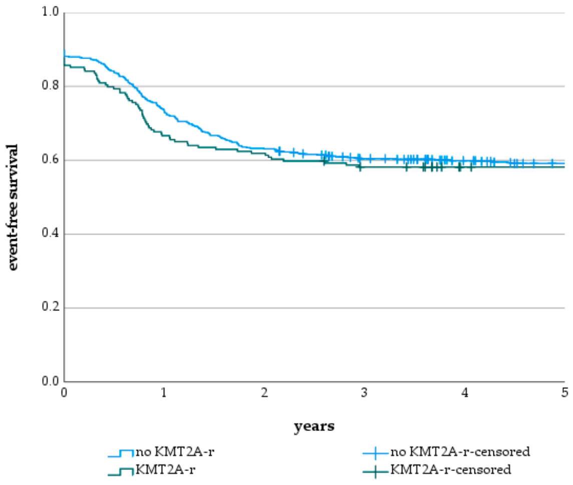

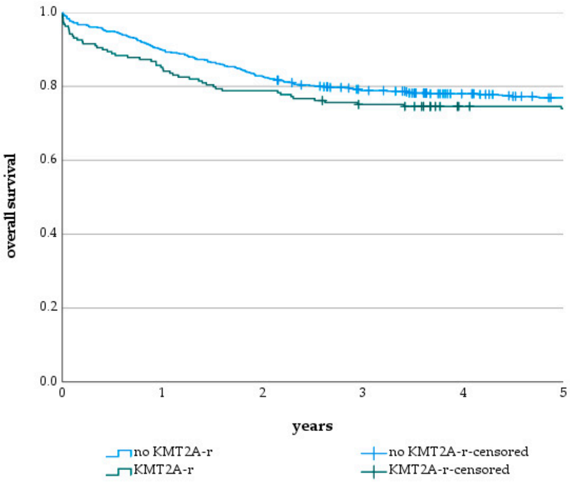

Copyright: © 2021 by the authors. Licensee MDPI, Basel, Switzerland. This article is an open access article distributed under the terms and conditions of the Creative Commons Attribution (CC BY) license (http://creativecommons.org/licenses/by/4.0/).

(**a**) (**b**)

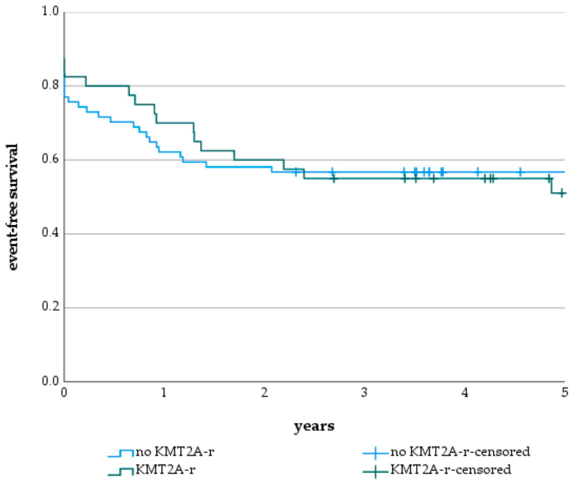

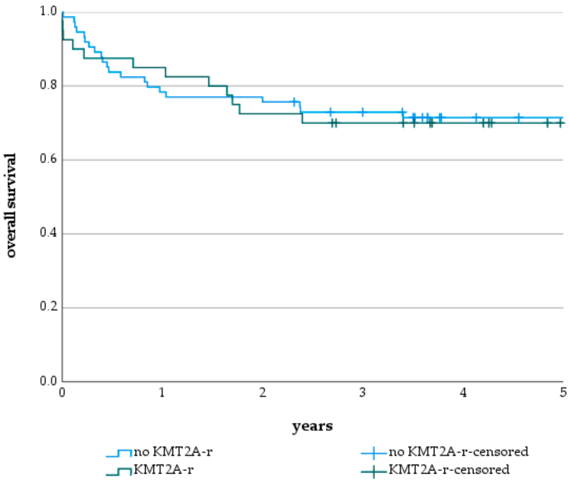

(**c**) (**d**)

Figure S1. Survival of pediatric AML patients depending on *KMT2A* rearrangements (*KMT2A*-r) and risk group. (**a**) 5-year event-free survival (EFS) of pediatric AML patients with *KMT2A*-r (58.2% ± 3.6%; n = 189) and without *KMT2A*-r (59.2% ± 2.0%; n = 607) stratified into standard or intermediate risk group (p = 0.523); (**b**) 5-year overall survival (OS) of pediatric AML patients with *KMT2A*-r (74.0% ± 3.2%; n = 189) and without *KMT2A*-r (76.9% ± 1.7%; n = 607) stratified into standard or intermediate risk group (p = 0.361); (**c**) 5-year event-free survival (EFS) of pediatric AML patients with *KMT2A*-r (51.1% ± 8.2%; n = 40) and without *KMT2A*-r (56.8% ± 5.8%; n = 74) stratified into high risk group (p = 0.864); (**d**) 5-year overall survival (OS) of pediatric AML patients with *KMT2A*-r (70.0% ± 7.2%; n = 40) and without *KMT2A*-r (71.5% ± 5.3%; n = 74) stratified into high risk group (p = 0.856). Significance was calculated with log-rank test.

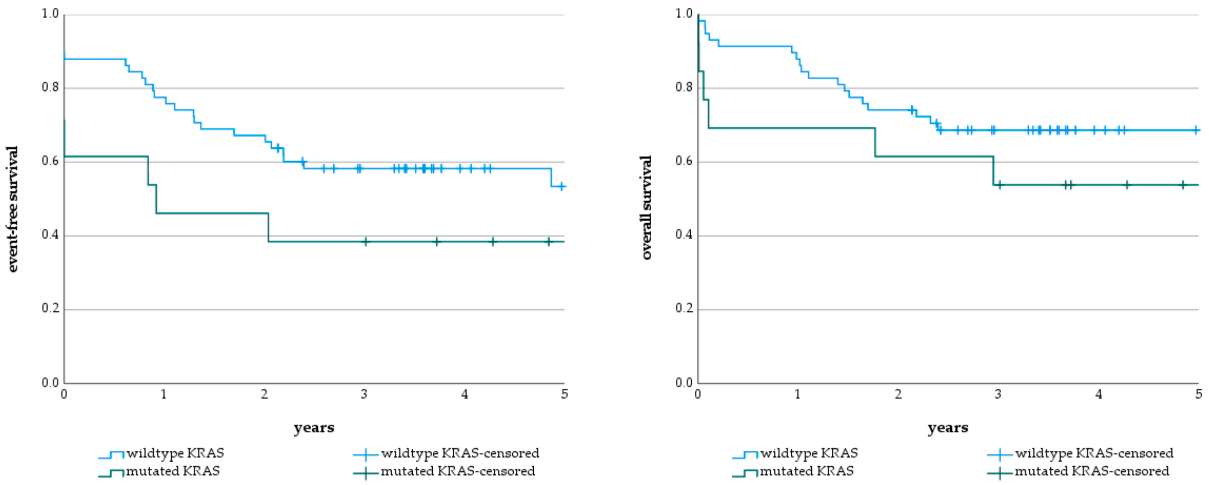

(**a**) (**b**)

Figure S2. Survival of pediatric AML patients with *KMT2A*-r and with or without *KRAS* mutation. (**a**) 5-year event-free survival (EFS) of pediatric AML patients with *KMT2A*-r and with wildtype (53.4% ± 7.6%; *n* = 58) or mutated *KRAS* (38.5% ± 13.5%; *n* = 13) (*p* = 0.113). (**b**) 5-year overall survival (OS) of pediatric AML patients with *KMT2A*-r and with wildtype (68.7% ± 6.1%; *n* = 58) or mutated *KRAS* (53.8% ± 13.8%; *n* = 13) (*p* = 0.103). Significance was calculated with log-rank test.

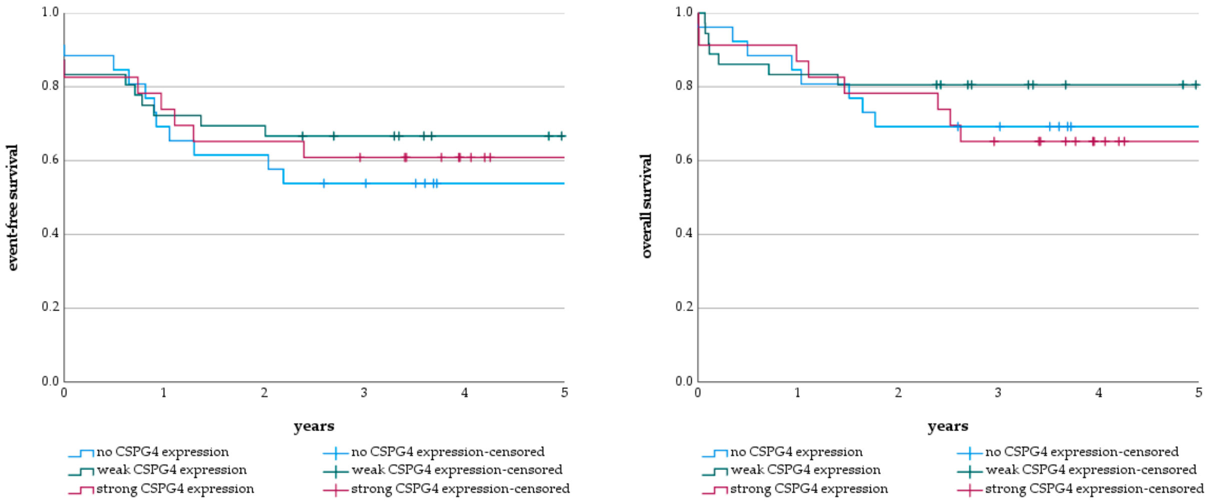

(**a**) (**b**)

Figure S3. Survival of pediatric AML patients with *KMT2A*-r depending on CSPG4 expression level. (**a**) 5-year event-free survival (EFS) of pediatric AML patients with *KMT2A*-r and without CSPG4 expression (53.8% ± 9.8%; *n* = 26), with weak (66.7% ± 7.9%; *n* = 36) or strong CSPG4 expression (60.9% ± 10.2%; *n* = 23) (*p* = 0.526). (**b**) 5-year overall survival (OS) of pediatric AML patients with *KMT2A*-r and without CSPG4 expression (69.2% ± 9.1%; *n* = 26), with weak (80.6% ± 6.6%; n=36) or strong CSPG4 expression (65.2% ± 9.9%; *n* = 23) (*p* = 0.398). Significance was calculated with log-rank test.

3. Supplementary Tables S1–S5

Table S1. Clinical characteristics of pediatric AML patients with and without mutational screening via next-generation sequencing using the Illumina TruSight Myeloid Panel (TSM).

Features		All Patients		TSM		No TSM		*p*-Value
Number		967	100%	309	32%	658	68%	
Age at diagnosis	Median	8.3		9.9		7.9		0.008 T (**)
	MIN	0.0		0.0		0.0		
	MAX	18.0		17.9		18.0		
Gender	Male	491	100%	168	34%	323	66%	0.126 C (n.s.)
	Female	476	100%	141	30%	335	70%	
WBC count [×10³/µl]	Median	16.1		22.7		13.5		0.010 T (*)
	MIN	0.0		0.6		0.0		
	MAX	817.1		817.1		585.0		
Hemoglobin [g/dl]	Median	8.3		8.3		8.3		0.706 T (n.s.)
	MIN	2.1		2.1		2.6		
	MAX	18.3		17.1		18.3		
Platelet count [×10³/µl]	Median	66.0		64.5		67.0		0.900 T (n.s.)
	MIN	2.0		6.0		2.0		
	MAX	1,370		722		1,370		
Risk groups	Standard risk	244	27%	54	21%	190	29%	<0.001 C (***)
	Intermediate risk	552	61%	133	52%	419	64%	
	High risk	114	13%	70	27%	44	7%	
	No data	57	-	52	-	5	-	-
Morphologic subtype (FAB classification)	M0	19	2%	5	2%	14	2%	0.052 C (n.s.)
	M1	112	14%	39	19%	73	12%	
	M2	175	22%	46	23%	129	21%	
	M4	134	17%	36	18%	98	16%	
	M4 eo	67	8%	16	8%	51	8%	
	M5	198	25%	48	24%	150	25%	
	M6	12	1%	2	1%	10	2%	
	M7	88	11%	11	5%	77	13%	
	No data	162	-	106	-	56	-	-
CSPG4 expression	Strong positive	31	9%	24	10%	7	6%	0.403 C (n.s.)
	Weak positive	55	16%	37	16%	18	16%	
	Negative	260	75%	170	74%	90	78%	
	No data	621	-	78	-	543	-	-

T unpaired *t*-test; C Pearson's chi square test; * $p < 0.05$; ** $p < 0.01$; *** $p < 0.001$; n.s. not significant

Table S2. Clinical characteristics of pediatric AML patients with and without analysis for CSPG4 expression via multicolor flow cytometry.

Features		All Patients		CSPG4 Cohort		Cohort Not Analyzed for CSPG4		*p*-Value
Number		967	100%	346	36%	621	64%	
Age at diagnosis	Median	8.3		9.1		7.9		0.101 [T] (n.s.)
	MIN	0.0		0.0		0.0		
	MAX	18.0		18.0		18.0		
Gender	Male	491	100%	174	35%	317	65%	0.821 [C]
	Female	476	100%	172	36%	304	64%	(n.s.)
WBC count [x10³/µl]	Median	16.1		19.5		14.2		0.687 [T] (n.s.)
	MIN	0.0		0.2		0.0		
	MAX	817.1		817.1		585.0		
Hemoglobin [g/dl]	Median	8.3		8.4		8.3		0.969 [T] (n.s.)
	MIN	2.1		2.1		2.6		
	MAX	18.3		17.1		18.3		
Platelet count [x10³/µl]	Median	66.0		66.0		66.0		0.753 [T] (n.s.)
	MIN	2.0		6.0		2.0		
	MAX	1370.0		863.0		1370		
Risk groups	Standard risk	244	27%	68	22%	176	29%	<0.001 [C] (***)
	Intermediate risk	552	61%	160	51%	392	66%	
	High risk	114	13%	85	27%	29	5%	
	No data	57	-	33	-	24	-	-
Morphologic subtype (FAB classification)	M0	19	2%	7	3%	12	2%	0.576 [C] (n.s.)
	M1	112	14%	34	14%	78	14%	
	M2	175	22%	56	22%	119	21%	
	M4	134	17%	48	19%	86	16%	
	M4 eo	67	8%	21	8%	46	8%	
	M5	198	25%	61	24%	137	25%	
	M6	12	1%	1	0%	11	2%	
	M7	88	11%	23	9%	65	12%	
	No data	162	-	95	-	67	-	-

[T] unpaired *t*-test; [C] Pearson's chi square test; * $p < 0.05$; ** $p < 0.01$; *** $p < 0.001$; n.s. not significant

Table S3. *KRAS* mutations and correlation with specific *KMT2A-r*.

	**mutated *KRAS***	**wildtype *KRAS***
no *KMT2A*-r	13	225
all AML with *KMT2A*-r	13	58
KMT2A/MLLT3	5	24
KMT2A/MLLT10	3	14
KMT2A/AFDN	1	2
KMT2A/MLLT1	0	6
KMT2A/MLLT11	0	2
KMT2A/AFF1	0	2
KMT2A/SEPTIN9	2	0
KMT2A/ELL	1	1
KMT2A/MLLT6	0	1
KMT2A/PRPF19	0	2
KMT2A/ABI2	0	1
KMT2A/KNL1	0	1
KMT2A/USP2	0	1
KMT2A-r not further specified	1	1

Table S4. Genetic aberrations and dependency on *KMT2A-r* status.

**Aberrations**	**no *KMT2A*-r (*n* = 238)**		***KMT2A*-r (*n* = 71)**	
	**Total***	**%**	**Total***	**%**
FLT3-ITD	64	26.9%	2	2.8%
WT1	50	21.0%	0	0.0%
NRAS	49	20.6%	17	23.9%
NUP98/NSD1	12 (of 65)	18.5%	0 (of 32)	0.0%
trisomy 8	32	13.4%	10	14.1%
NPM1	28	11.8%	0	0.0%
RUNX1/RUNX1T1	27	11.3%	0	0.0%
CBFB/MYH11	23	9.7%	0	0.0%
CEBPA	23	9.7%	0	0.0%
KIT	14	5.9%	0	0.0%
KRAS	13	5.5%	13	18.3%
MLLT10/PICALM	3 (of 56)	5.4%	0 (of 32)	0.0%
PICALM/MLLT10	3 (of 56)	5.4%	0 (of 32)	0.0%
monosomy 7	12	5.0%	1	1.4%
FLT3-TKD	11	4.6%	6	8.5%
PTPN11	10	4.2%	1	1.4%
ASXL1	9	3.8%	2	2.8%
GATA2	9	3.8%	0	0.0%
MIR3667HG/BRD1	2 (of 56)	3.6%	0 (of 32)	0.0%
KAT6A/CREBBP	2 (of 56)	3.6%	0 (of 32)	0.0%
PRDM16/SKI	2 (of 56)	3.6%	0 (of 32)	0.0%
RUNX1/CBFA2T3	2 (of 229)	3.6%	0 (of 69)	0.0%
RUNX1	7	2.9%	0	0.0%
TET2	6	2.5%	0	0.0%
DNMT3A	5	2.1%	0	0.0%
IDH2	5	2.1%	0	0.0%
CBFB/NFATC3	1 (of 56)	1.8%	0 (of 32)	0.0%
CPD/LRRC37B	1 (of 56)	1.8%	0 (of 32)	0.0%
ETV6/MN1	1 (of 56)	1.8%	0 (of 32)	0.0%
ETV6/NCOA2	1 (of 56)	1.8%	0 (of 32)	0.0%
ETV6/NDUFA5	1 (of 56)	1.8%	0 (of 32)	0.0%
G3BP1/PDGFRB	1 (of 56)	1.8%	0 (of 32)	0.0%
KDM5A/NUP98	1 (of 56)	1.8%	0 (of 32)	0.0%
MN1/ETV6	1 (of 56)	1.8%	0 (of 32)	0.0%
MTCH2/WT1	1 (of 56)	1.8%	0 (of 32)	0.0%
NCOA2/ETV6	1 (of 56)	1.8%	0 (of 32)	0.0%
NPM1/MLF1	1 (of 56)	1.8%	0 (of 32)	0.0%
NUP214/ABL1	1 (of 56)	1.8%	0 (of 32)	0.0%
NUP98/KDM5A	1 (of 56)	1.8%	0 (of 32)	0.0%
PIM3/BRD1	1 (of 56)	1.8%	0 (of 32)	0.0%
PSPC1/ZMYM2	1 (of 56)	1.8%	0 (of 32)	0.0%
SLC17A5/PICALM	1 (of 56)	1.8%	0 (of 32)	0.0%
SPI1/ZNF384	1 (of 56)	1.8%	0 (of 32)	0.0%
UGCG/PVT1	1 (of 56)	1.8%	0 (of 32)	0.0%
ZNF384/SPI1	1 (of 56)	1.8%	0 (of 32)	0.0%
DEK/NUP214	4 (of 229)	1.7%	0 (of 69)	0.0%
BCORL	4	1.7%	1	1.4%
ETV6/TEL	4	1.7%	1	1.4%
EZH2	3	1.3%	3	4.2%
JAK2	3	1.3%	0	0.0%
KDM6A	3	1.3%	0	0.0%
PHF6	3	1.3%	0	0.0%
RAD21	3	1.3%	2	2.8%

TP53	3	1.3%	1	1.4%
BCOR	2	0.8%	1	1.4%
IDH1	2	0.8%	0	0.0%
KMT2A	2	0.8%	0	0.0%
NOTCH1	2	0.8%	0	0.0%
trisomy 12	2	0.8%	0	0.0%
CBFA2T3/GLIS2	1 (of 230)	0.4%	0 (of 69)	0.0%
BCR/ABL	1	0.4%	0	0.0%
CBL	1	0.4%	0	0.0%
CDKN2A	1	0.4%	1	1.4%
CSF3R	1	0.4%	0	0.0%
IKZF1	1	0.4%	0	0.0%
MPL 1	1	0.4%	0	0.0%
PTEN	1	0.4%	0	0.0%
SMC1A	1	0.4%	0	0.0%
STAG2	1	0.4%	0	0.0%
U2AF1	1	0.4%	1	1.4%
ZRSR2	1	0.4%	0	0.0%
ABL1	0	0.0%	0	0.0%
ARHGAP21/KMT2A	0 (of 56)	0.0%	1 (of 32)	3.1%
ATRX	0	0.0%	0	0.0%
BRAF	0	0.0%	0	0.0%
CALR	0	0.0%	0	0.0%
CBLB	0	0.0%	0	0.0%
CBLC	0	0.0%	0	0.0%
CUX1	0	0.0%	0	0.0%
ELL/KMT2A	0 (of 56)	0.0%	1 (of 32)	3.1%
FBXW7	0	0.0%	0	0.0%
GATA1	0	0.0%	0	0.0%
GNAS	0	0.0%	0	0.0%
HRAS	0	0.0%	0	0.0%
JAK3	0	0.0%	0	0.0%
MLLT10/CCNYL1	0 (of 56)	0.0%	1 (of 32)	3.1%
MLLT10/DNAJC1	0 (of 56)	0.0%	1 (of 32)	3.1%
MLLT10/GUCY1A2	0 (of 56)	0.0%	1 (of 32)	3.1%
MLLT10/LOC100131626	0 (of 56)	0.0%	1 (of 32)	3.1%
MLLT10/PIP4K2A	0 (of 56)	0.0%	1 (of 32)	3.1%
MLLT10/SDHAF2	0 (of 56)	0.0%	1 (of 32)	3.1%
MLLT10/SFMBT2	0 (of 56)	0.0%	1 (of 32)	3.1%
MLLT10/ZMYM2	0 (of 56)	0.0%	1 (of 32)	3.1%
MLLT1/KMT2A	0 (of 56)	0.0%	2 (of 32)	6.3%
MLLT3/KMT2A	0 (of 56)	0.0%	1 (of 32)	3.1%
MLLT6/KMT2A	0 (of 56)	0.0%	1 (of 32)	3.1%
MYD88	0	0.0%	0	0.0%
PDGFRA	0	0.0%	0	0.0%
PIP4K2A/KMT2A	0 (of 56)	0.0%	1 (of 32)	3.1%
PIWIL4/ZMYM2	0 (of 56)	0.0%	1 (of 32)	3.1%
SETBP1	0	0.0%	0	0.0%
SF3B1	0	0.0%	0	0.0%
SMC3	0	0.0%	0	0.0%
SRSF2	0	0.0%	0	0.0%
ZMYM2/TAS2R13	0 (of 56)	0.0%	1 (of 32)	3.1%

*If the number of analyzed patients differentiates from the total cohort, the number of analyzed patients is stated in parentheses.

Table S5. Samples of patients with weak or strong CSPG4 expression and associated gene fusions and mutations.

	ID	*KAT6A/NCOA2*	*NUP98/KDM5A*	*KAT6A/EP300*	*KAT6A/CREBBP*	*CBFB/MYH11*	*KDM6A*	*WT1*
weak CSPG4 expression	AMLR12DEKR4	-	-	-	-	-	-	-
	AMLR12DEEF6	-	-	-	-	-	-	-
	AMLR12DEHV9	-	positive	-	-	-	-	-
	AMLR12DEF 5	positive	-	-	-	-	-	-
	AMLR12DES 9	-	-	-	-	-	positive	-
	AMLR12DEMS4	-	-	-	-	-	-	-
	AMLR12DEK 2	-	-	-	-	-	-	-
	AML-4 DEBI17	-	-	-	-	-	-	positive
	AMLS12DEE 2	-	-	-	-	positive	-	-
	AMLR12DEE 5	-	-	-	-	positive	-	-
	AMLR12DEFB4	-	-	-	-	positive	-	positive
	AMLR12DERE2	-	-	-	-	positive	-	-
	AMLS12DET 2	-	-	-	-	positive	-	-
	AMLS12DEHV2	-	-	-	-	positive	-	-
	AMLR17DEGI4	-	-	-	-	-	-	-
	AMLS12DES 1	-	-	-	-	positive	-	positive
	AMLR17DEMZ2	-	-	-	-	positive	-	-
	AMLS12DEDR6	-	-	-	-	positive	-	-
	AMLR12DEWB4	-	-	-	-	positive	-	-
strong CSPG4 expression	AMLR12DEBI3	-	-	-	-	-	-	-
	AMLR12DEM 14	-	-	positive	-	-	-	-
	AMLR12DEBI1	-	-	-	-	-	-	positive
	AMLR12DEM 24	-	-	-	positive	-	-	-
	AMLR17DES 9	-	-	-	-	-	-	-
	AMLR17DES 6	-	-	-	-	-	-	-
	AMLR17DEHH2	-	-	-	-	-	-	-
	AMLS12DES 3	-	-	-	-	-	-	-
